# Gene expression time-series analysis of Camptothecin effects in U87-MG and DBTRG-05 glioblastoma cell lines

**DOI:** 10.1186/1476-4598-7-66

**Published:** 2008-08-11

**Authors:** Elena Morandi, Cinzia Severini, Daniele Quercioli, Giovanni D'Ario, Stefania Perdichizzi, Miriam Capri, Giovanna Farruggia, Maria Grazia Mascolo, Wolfango Horn, Monica Vaccari, Roberto Serra, Annamaria Colacci, Paola Silingardi

**Affiliations:** 1 Excellence Environmental Carcinogenesis, Lab. Mater, Environmental Protection and Health Prevention Agency – Emilia-Romagna Region (ER-EPA), Viale Filopanti 22, 40126, Bologna County, Italy; 2 The FIRC Institute of Molecular Oncology Foundation, Via Adamello 16, 20139, Milan, Italy; 3 Department of Experimental Pathology-Cancer Research Section, University of Bologna, Viale Filopanti 22, 40126, Bologna, Italy; 4 CIG, Interdepartmental Research Centre "L. Galvani", University of Bologna, Via S. Giacomo 12, 40126, Bologna, Italy; 5 Department of Biochemistry, University of Bologna, Via Irnerio 48, 40126, Bologna, Italy; 6 Department of Social, Cognitive and Quantitative Sciences, University of Modena and Reggio Emilia, Via Allegri 9, Reggio Emilia, Italy

## Abstract

**Background:**

The clinical efficacy of camptothecin (CPT), a drug specifically targeting topoisomerase I (TopoI), is under evaluation for the treatment of malignant gliomas. Due to the high unresponsiveness of these tumours to chemotherapy, it would be very important to study the signalling network that drives camptothecin outcome in this type of cancer cells. To address this issue, we had previously compared the expression profile of human U87-MG glioblastoma cells with that of a CPT-resistant counterpart, giving evidence that the development of a robust inflammatory response was the main transcriptional effect associated with CPT resistance.

Here we report time-related changes and cell line specific patterns of gene expression after CPT treatment by using two p53 wild-type glioblastoma cell lines, U87-MG and DBTRG-05, with different sensitivities to TopoI inhibition.

**Results:**

First, we demonstrated that CPT treatment brings the two cell lines to completely different outcomes: accelerated senescence in U87-MG and apoptosis in DBTRG-05 cells. Then, to understand the different susceptibility to CPT, we used oligo-microarray to identify the genes whose expression was regulated during a time-course treatment, ranging from 2 h to 72 h. The statistical analysis of microarray data by MAANOVA (MicroArray ANalysis Of VAriance) showed much less modulated genes in apoptotic DBTRG-05 cells (155) with respect to the senescent U87-MG cells (3168), where the number of down-regulated genes largely exceeded that of the up-regulated ones (80% vs. 20%). Despite this great difference, the two data-sets showed a large overlapping (60% circa) mainly due to the expression of early stress responsive genes. The use of High-Throughput GoMINER and EASE tools, for functional analysis of significantly enriched GO terms, highlighted common cellular processes and showed that U87-MG and DBTRG-05 cells shared many GO terms, which are related to the down-regulation of cell cycle and mitosis and to the up-regulation of cell growth inhibition and DNA damage.

Furthermore, the down-regulation of MYC and DP1 genes, which act as key transcription factors in cell growth control, together with the inhibition of BUB1, BUB3 and MAD2 mRNAs, which are known to be involved in the spindle checkpoint pathway, were specifically associated with the execution of senescence in U87-MG cells and addressed as critical factors that could drive the choice between different CPT-inducible effectors programs. In U87-MG cells we also found inflammation response and IL1-beta induction, as late transcriptional effects of Topo I treatment but these changes were only partially involved in the senescence development, as shown by IL1-beta gene silencing.

**Conclusion:**

By comparing the transcription profile of two glioblastoma cell lines treated with camptothecin, we were able to identify the common cellular pathways activated upon Topo I inhibition. Moreover, our results helped in identifying some key genes whose expression seemed to be associated with the execution of senescence or apoptosis in U87-MG and DBTRG-05 cells, respectively.

## Background

Camptothecin, a naturally occurring cytotoxic alkaloid, and its water-soluble derivatives belong to a family of antineoplastic agents specifically targeting topoisomerase I. They exert their main S-phase cytotoxic activity through the accumulation of DNA double-strand breaks, originating from the collision between the replication fork and the ternary complex TopoI-CPT-DNA [1,2]. In general, CPT treatment arrests cells in G2 phase and can trigger rapid apoptosis in some cell types [3]. However, in the presence of low CPT doses and limited DNA damage extension, the induction of a senescence-like-phenotype (SLP) was observed [4-6]

Most of the molecular events elicited by CPT deal with stress response and cell survival signaling pathways activated by DNA damage [7,8]. Beside the ATM/Chk2 DNA damage checkpoint pathway, the ATR-Chk1 response has recently been described to play a predominant role in the response to TopoI inhibition [9]. Furthermore, in many cellular systems, CPT was able to activate NF-kB, a key transcriptional factor that regulates the survival response induced by many chemotherapeutics [10,11]. All these pathways clearly converge on the transcriptional machinery, thus affecting the transcriptome in a relevant manner. For this reason, a global analysis of gene expression modulation by microarray could produce new insights into the complexity of TopoI poisons stress responses [12].

The transcriptional profile of CPT effects has been determined in HCT116 synchronized human colon cancer cells, in a time-series study, by treating cells with relatively low (20 nM) and high (1000 nM) drug concentrations [13]. A microarray analysis was also reported for HeLa cells and ML-2 myeloid leukemia cells, in a single-time treatment experiment with a CPT concentration able to induce apoptosis [3,14]. Recently, the changes in gene expression, following the short-term exposure of HL60 cells, to SN-38, the active metabolite of camptothecin, were compared with those derived from the peripheral blasts of patients with acute and chronic myeloid leukemia, that had undergone a therapy with a single dose of Irinotecan (CPT-11), a water soluble derivative of camptothecin [15]. This study showed a number of genes whose expression was commonly affected by CPT-11 both *in vitro *and *in vivo*. In addition, Reinhold *et al*. investigated the mechanism of CPT-11 resistance in DU145 human prostate cancer cells, by using microarray technology and analysis [16].

All these studies were useful to generate new hypothesis on the mechanisms of Topo1 inhibition that were unpredictable on the basis of the known properties of the drug.

As the use of camptothecins is under evaluation for the treatment of malignant gliomas [17,18], we previously analysed the transcriptional profile of CPT resistant U87-MG glioma cells, obtained from repeated exposures of the parental cell line to a high CPT concentration [19]. Our results had demonstrated that many of the most up-regulated genes trigger cellular mechanisms, like inflammatory response and angiogenesis, that can negatively impact upon chemotherapy efficacy.

To clarify the effects of TopoI inhibition in glioblastoma cells, in the present study we first checked the sensitivity of two human glioblastoma cell lines, U87-MG and DBTRG-05, to CPT and, then, we characterized the CPT-induced transcriptional response during a short time-course treatment ranging from 2 to 72 hours, by using high density oligonucleotide (60-mer) array technology. Although the two cell lines are both representative of high grade glioblastoma and are considered p53 wild-type, DBTRG-05 cells underwent mainly apoptosis while U87-MG cells developed a senescence-like phenotype.

The expression profiling showed the common biological processes that are modulated by TopoI in both cell lines and identified specific gene networks linked to the development of senescence in U87-MG cells. Apoptosis and senescence are considered two alternative mechanisms of the chemotherapy-induced tumor repression. Many questions, however, still, remain open about the effective role that accelerated senescence has in a clinical setting, mainly due to its potential detrimental effects [20-22]. Therefore, the transcriptional analysis of the mechanisms underlying the execution of two distinct camptothecin effectors programs could help in better understanding the final outcome of TopoI poisoning.

## Results

### CPT treatment promotes a senescence-like phenotype in U87-MG cells but induces apoptosis in DBTRG-05 cells

**Figure 1:**
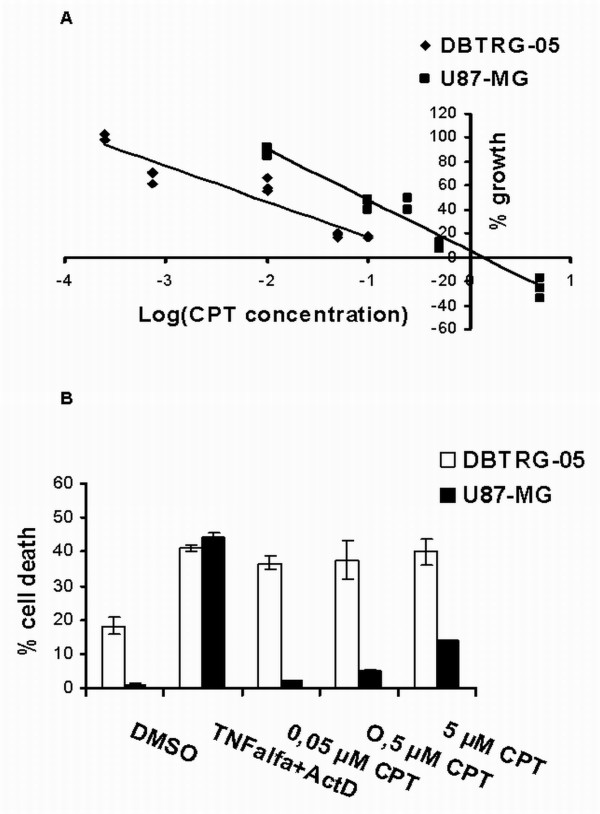
**CPT-induced acute citotoxicity and apoptosis**. **A**. Dose-response analysis of CPT effects on U87-MG and DBTRG-05 cells. On Y-axis the percentage of growth, calculated according to the protocol used by the National Cancer Institute, is reported. The GI50 values for both cell lines were calculated by linear regression analysis of the log (dose/effect) curve. **B**. Annexin-V apoptosis assay was performed after a 24-h treatment with 0.5 and 5 μM CPT. DMSO and TNF-alpha plus actinomycin D were used as negative and positive controls, respectively. Each data point is the average of three independent experiments; bars, standard errors.

The effect of CPT on glioblastoma cells was first assessed by the crystal violet growth inhibition assay. The dose-response curves from a 72-hour treatment with different concentrations of CPT, were used to calculate the GI50 values (50% Growth Inhibition). As shown in Figure 1A, DBTRG-05 cells are more sensitive to TopoI inhibition, being the GI50 value five times lower in this cell line (0.018 μM) than in U87-MG cells (0.09 μM).

**Figure 2:**
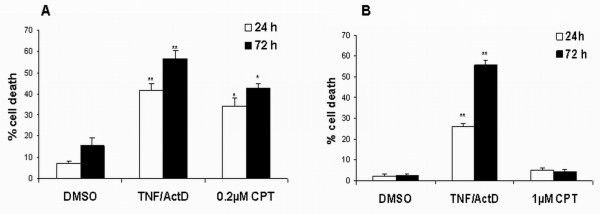
**Apoptotic response of DBTRG-05 and U87-MG cells exposed to CPT working concentrations**. **A**. Percentages of Annexin-V positive DBTRG-05 cells treated for 24 and 72 hours with 0.2 μM CPT. * p < 0.05, Student's t-test. ** p < 0.01, Student's t-test. B. Percentages of Annexin-V positive U87-MG cells treated for 24 and 72 hours with 1 μM CPT; bars, standard errors. ** p < 0.01, Student's t-test.

The ability of CPT to induce apoptosis was then analyzed in both cell lines, by using the Annexin-V method (Figure 1B). Even if the positive control (actinomycin D plus TNF alpha) triggered a similar response in the two cell lines, only DBTRG-05 cells showed a significant induction of apoptosis, after 24 h-treatment with CPT, even at the lower assayed dose of 0.05 μM. U87-MG cells remained substantially refractory to apoptosis, at all the assayed concentrations except for a slight effect (13.9%) detected at the dose 5 μM CPT. These data were further confirmed by using CPT at the concentrations of 0.2 μM and 1 μM, for DBTRG-05 and U87-MG cells, respectively (Figure 2). These doses were extrapolated from the linear regression curves, as showed in Figure 1A, to give a similar growth inhibitory effect, on both cell lines, with a maximum effect (100% growth inhibition) after 72-hours treatment. Camptothecin was able to induce a significant increase of apoptosis in DBTRG-05 cells, after 24 and 72 hours treatments, while no up-regulation of annexin-V positive cells was observed in the U87-MG population (Figure 2A and 2B).

**Figure 3:**
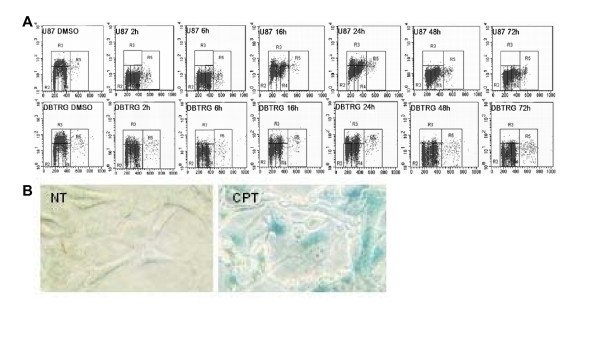
**BrdU Flow cytometry assay of U87-MG and DBTRG-05 cells exposed to CPT and SA-β Gal staining in treated U87-MG cells**. **A**. Time dependent changes in DNA content following treatment, from 2 to 72 hours, of U87-MG and DBTRG-05 cells with 1 μM and 0.2 μM CPT, respectively. DMSO: solvent control **B**. SA-β Gal staining of CPT-exposed U87-MG cells (CPT) versus control (NT). For this purpose, a 72 h treatment with 1 μM CPT was performed. Counts were made on three random fields of about 100 cells.

The cell cycle distribution of the two cell lines, in response to CPT treatment, was then assessed by using flow cytometry after pulse-BrdU incorporation. DNA TopoI inhibition caused almost similar effects on the two cell lines, during a time-course treatment ranging from 2 to 72 hours (Figure 3A). Both U87-MG and DBTRG-05 cells underwent a considerable G2/M arrest (U87 72 h/U87 NT: 27.9%/19.6%; DBTRG 72 h/DBTRG NT:33.8%/12.6%) with a concomitant increase in the percentage of non-cycling S-phase cells at all the tested time intervals. A slight increase in the population of cells with a DNA content above 4N (6.6%) was observed in U87-MG cells 72 hours after CPT exposure. The apparent recovery of BrdU-positive cells, that we observed after 16 h and 24 h from the treatments, was associated to the aspecific fluorescence caused by CPT, as shown by the shift to the right of the 16 h- and 24 h-S phase monoparametric curves and by the microscope analysis of cells under fluorescence (data not shown). The FACS data, together with the observation that U87-MG cells became flatter with enlarged nuclei in response to CPT, suggested that TopoI inhibition could elicit a senescence-like phenotype in this cell line. We then investigated the expression of senescence-associated beta-galactosidase activity (SA-β-gal) and showed that U87-MG cells stained significantly positive for SA-β-Gal, after a 72-hours treatment with 1 μM CPT (Figure 3B).

### Microarray analysis of time course treatment with CPT

To better characterize the molecular mechanisms underlying the response to CPT, we next analyzed the changes in gene expression, during a time-course treatment ranging from 2 to 72 hours, with the drug concentrations of 0.2 μM for DBTRG-05 cells and 1 μM for U87-MG cells. For this purpose, we used high density oligonucleotide (60 mer) microarrays containing more than 17.000 unique probes (Human1A oligo microarray, G4110A, Agilent Technologies, Palo Alto, CA).

The statistical analysis of the two time-series data sets was performed with MAANOVA, a collection of functions for the statistical analysis of gene expression data from two-color cDNA microarray experiments that is implemented as an add-on package for the freely available and widely used statistical language/software R (see details in materials and methods) [23].

**Figure 4:**
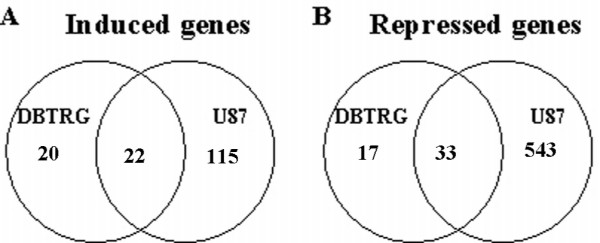
**Venn-diagram representation of gene transcripts up- (A) and down-regulated (B) by CPT in either/both U87-MG and DBTRG-05 cell lines**.

**Figure 5:**
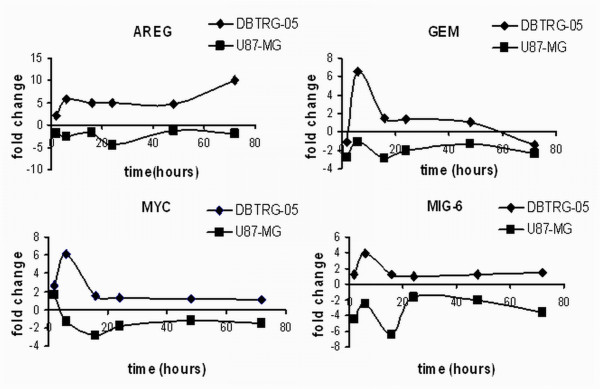
**Time-dependent expression profile of AREG, GEM, MYC and MIG6**. Log_2 _(expression ratio) was plotted versus time.

**Figure 6:**
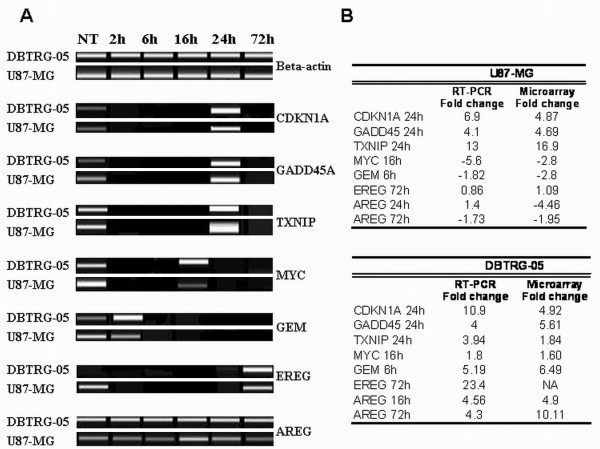
**Semi-quantitative RT-PCR post-validation of microarray results**. **A**. Gene expression changes for seven genes (CDKN1A, GADD45, TXNIP, MYC, GEM, AREG, EREG) were measured at selected time points after CPT treatment by semi-quantitative RT-PCR and normalized to β-actin expression of time- and dose- matched controls. The RT-PCR reactions were analyzed with the DNA1000 kit of the Agilent 2100 Bioanalyzer to give a gel-like image. **B**. At each selected time point after CPT treatment, expression fold change values obtained with semi-quantitative RT-PCR and microarrays were compared.

The lists of differentially expressed genes were numerically completely different. Applying a p-value threshold of 0.01 and 0.05 for U87-MG and DBTRG-05 cells respectively, the total number of differentially expressed genes was 3168 in the U87- and only 155 in the case of the DBTRG-05. To facilitate the comparison between the two data-sets, we restricted the analysis to those genes that reached a log_2 _(expression ratio) value above 1.5, at least in one of the six time-series data points. By doing that, we reduced the overall number of differentially expressed genes to 713 for U87-MG cells and to 92 for DBTRG cells (see Additional files 1 and 2 to retrieve the complete lists of genes returned from MAANOVA analysis and the filtering process). Based on the time dependent expression profile, we defined the up- and down-regulated genes within the two data sets. Interestingly, this analysis, that is summarized in the Venn diagrams reported in Figure 4, demonstrated that most of the genes, whose expression was affected by the treatment with CPT, were indeed down-regulated (almost 80%) in U87-MG cells, while a substantial balance between up- and down-regulation was observed in the DBTRG-05. Despite this difference, the two data-sets were largely overlapping, being more than the 60% of the genes found in DBTRG-05 cells also represented in the U87-MG population. In Table 1 and Table 2, we reported the lists of the common up-regulated genes and the down-regulated ones, respectively. Only four genes (AREG, MYC, MIG-6 and GEM) showed a different time-dependent expression profile between the two data-sets (Figure 5). Indeed, CPT treatment induced the expression of these genes in DBTRG-05 cells while repressed them in U87-MG cells. For selected time-points, three of these genes (AREG, MYC, GEM) were chosen to be confirmed through a semi-quantitative RT-PCR analysis, together with other genes (CDKN1A, TXNIP, GADD45A, EREG) known to be CPT transcriptional targets (Figure 6). Taking into account that we used a semi-quantitative approach, we found overlapping results between two techniques except for AREG in U87- and EREG in DBTRG-05 cells whose over-expression was undetectable in microarray.

**Table 1:** List of Genes up-regulated following CPT treatment in both U87-MG and DBTRG-05 cells.

**GeneName**	**Description**
	**DNA-Damage and/or stress-responsive genes**
ATF3	*Activating transcription factor 3*
CDKN1A	*Cyclin-dependent kinase inhibitor 1A*
DDIT3	*DNA damage inducible transcript 3*
DUSP5	*Dual specificity phosphatase 5*
EGR1	*Early growth response 1*
EGR3	*Early growth response 3*
FDXR	*Ferredoxin reductase*
GADD45A	*DNA damage inducible transcript 1*
p53CSV	*p53-inducible cell-survival factor*
PIG3	tumor protein p53 inducible protein 3
SAT	*Spermidine/spermine N1-acetyltransferase*
TIEG	*TGFB inducible early growth response*
TRIB3	*tribbles homolog 3 (Drosophila)*
	**miscellaneous**
GA	*Breast cell glutaminase*
I_1201768	*Protein of unknown function*
I_928877	*Protein of unknown function*
I_929113	*Protein with very strong similarity to ribonucleotide reductase induced by p53 (human p53R2)*
IL8	*Interleukin 8*
KLF5	*Kruppel-like factor 5 (intestinal)*
PPIF	*Peptidylprolyl isomerase F*
SEI1	*Cyclin-dependent kinase 4-binding protein*
EDIL3	*EGF-like repeats and discoidin I-like domains 3*

**Table 2:** Genes down-regulated following CPT treatment in both U87-MG and DBTRG-05

***GeneName***	***Description***
	**DNA metabolism and replication**
ADK	*Adenosine kinase*
CDC45L	*Cell division cycle 45 like*
DTYMK	*Deoxythymidylate kinase (dTMP kinase)*
FEN1	*Flap structure specific endonuclease 1*
H2AFZ	*H2A histone family Z*
KIAA0101	*The PCNA-associated factor KIAA0101/p15(PAF)*
MCM2	*Mini chromosome maintenance deficient 2*
MCM3	*Minichromosome maintenance deficient 3*
MCM7	*Minichromosome maintenance deficient 7*
RRM1	*Ribonucleotide reductase M1 subunit*,
RRM2	*Ribonucleotide reductase subunit M2*,
TYMS	*Thymidylate synthetase*
UHRF1	*Nuclear protein 95*
	**Cell cycle and mitosis**
*ANKT*	*nucleolar and spindle associated protein 1*
BIRC5	*Survivin*
CCNA2	*Cyclin A2*
CDC2	*Cell division cycle 2*
CDCA5	*cell division cycle associated 5*
KNSL6	*Mitotic centromere-associated kinesin (kinesin-like 6)*
PRC1	*Protein regulator of cytokinesis 1*
STMN1	*Stathmin 1 (oncoprotein 18)*
TOPK	*PDZ-binding kinase*
UBE2C	*Ubiquitin-conjugating enzyme E2C*
ZWINT	*ZW10 interactor*
	**Miscellaneous**
DKFZp762E1312	*Protein of unknown function*
I_932099	*Protein with high similarity to human HNRPA1*,
ID1	*Inhibitor of DNA binding 1*
KPNA2	*Karyopherin alpha 2 (importin alpha 1)*,
LOXL2	*Lysyl oxidase-like 2*
p100	*EBNA-2 co-activator (100 kD)*
SPOCK	*Testican*
TSSC3	*Tumor suppressing subtransferable candidate 3*
UBE2T	*ubiquitin-conjugating enzyme E2T*

### Functional analysis of microarray data through High-Throughput GoMINER and EASE

To have a general picture of the most affected biological processes during the time-course treatment, as defined by Gene Ontology annotation, first we used the High-Throughput GoMINER analysis tool [24]. This package allows the automatic analysis of multiple microarrays and then integrates the results, across all of them, to find the GO categories that were significantly over-represented. To reach this goal the program first estimates the p-value using Fisher's exact test and then corrects the p-values for the multiple comparisons by estimating the FDR (False Discovery Rate).

For each time point, we defined as up-regulated or down-regulated those genes having a log_2_(expression ratio) above 1.5. The results were represented as clustered image maps (CIM), where the enriched GO categories (FDR < 0.05) are hierarchically clustered (Euclidean distance, average linkage clustering) against time either for the up- and the down- regulated genes. (Additional file 3, 4 and 5).

Two main blocks of biological processes were down-regulated in both cell lines treated with CPT. The first was DNA metabolism (GO:0006260), with all the correlated molecular mechanisms regulating dNTPs metabolism, DNA replication and repair, and the second was mitosis and all the events regarding the regulation of M-phase (GO:0000087) as well as the formation and maintenance of the spindle (GO:0007051_spindle_organization_and_biogenesis). Most of the above biological processes converged into the main down-regulated process, that was cell cycle (GO:0007049). In Table 3, we listed the genes belonging to some of the more representative down-regulated GO categories.

**Table 3:** List of genes belonging to some of the most significantly down-regulated GO categories as resulted by GoMiner Hightroughput analysis

**Cell Cycle**		**DNA metabolism**		**M-Phase**	
**U87**	**DBTRG**	**U87**	**DBTRG**	**U87**	**DBTRG**

CDC45L	MCM7	CDC6	UBE2C	POT1	MCM7
MCM3	CDC45L	PRC1	CDC2	CDC45L	CDC45L
PRC1	MCM3	BUB1	STMN1	MCM3	RRM2
TFDP1	PRC1	MAD2L1	PRC1	FEN1	MCM3
CKS2	TOPK	TOPK	KPNA2	PRIM2A	KPNA2
MAD2L1	UHRF1	CCNA2	TOPK	EZH2	FEN1
EXT1	CCNA2	KNSL5	CCNA2	USP1	UHRF1
CCNB2	CDC2	UBE2C		RFC3	DTYMK
CDKN2D	UBE2C	CCNB2		RAD54B	RRM1
RAD54B	ZWINT	CDC2		TOP2A	TYMS
BIRC5	STMN1	STMN1		MCM6	MCM2
MCM6	KPNA2	KPNA2		TYMS	
TTK	BIRC5	PKMYT1		NFIB	
CDKN3	DTYMK	RAD54B		MID1	
TOPBP1	MCM2	CCNB1		TOPBP1	
CDC6		TTK		CDC6	
MCM7		SMC2L1		PRKDC	
MNAT1				MCM7	
DLG1				MNAT1	
BUB1				RFC5	
UHRF1				UHRF1	
NOTCH2				RRM1	
TOPK				HMG1	
CCNA2				RRM2	
KNSL5				NT5E	
CDC2				KPNA2	
UBE2C				MSH2	
BARD1				HMG2	
ZWINT				DTYMK	
CKS1				MCM2	
MKI67					
STMN1					
MSH2					
KPNA2					
PKMYT1					
CCNB1					
RB1					
DTYMK					
CCNE2					
MCM2					
SMC2L1					

Between the up-regulated GO terms, the response to DNA-damage stimulus (GO:0006974) and cell cycle arrest (GO:0007050) appeared to be the biological processes that were more significantly affected by CPT in both cell line.

The two data sets were also analyzed by EASE, a publicly available and stand-alone application for use on Windows operating systems [25]. We used EASE to identify the enriched GO-Categories corresponding to "biological processes" in the list of up and down-regulated genes with respect to the whole list of genes of the microarray. In Table 4 we reported only those categories having an EASE score, corrected for multiple comparisons with the Bonferroni method, below 0.05.

**Table 4:** List of the biological processes as defined by the Gene Ontology that resulted enriched in the list of up-redulated and down-regulated genes in U87-MG and DBTRG-05 by EASE analysis (EASE score, Bonferroni < 0.05)

**U87 DOWN**	**DBTRG DOWN**
*Cell cycle*	*Mitotic cell cycle*
*Mitotic cell cycle*	*Cell cycle*
*Cell proliferation*	*Cell proliferation*
*Cell growth and maintenance*	*DNA metabolism*
*Cellular process*	*Deoxyribonucleic acid metabolism*
*DNA replication and chromosome cycle*	
*DNA replication*	
*S-phase of mitotic cell cycle*	
*Mitosis*	
*M-phase of mitotic cell cycle*	
*DNA metabolism*	
*DNA dependent DNA replication*	
*Cell cycle checkpoints*	
*M-phase*	
*Nuclear division*	
*G1-S transition of mitotic cell cycle*	

**U87 UP**	**DBTRG UP**

*Negative regulation of cell proliferation*	*N.A*.
*Regulation of cellular process*	
*Regulation of biological process*	
*Regulation of cell proliferation*	
*Response to external stimulus*	
*Cell proliferation*	
*Response to stress*	

Even if EASE analysis was more restrictive than that of High-Throughput GoMINER (also due to the choice of the stringent Bonferroni correction), it gave "cell cycle" and "DNA metabolism" as common CPT-down-regulated biological processes in the two glioblastoma cell lines.

No significant enriched GO category was found by EASE in the list of up-regulated DBTRG genes whereas in U87-MG cells, besides the "negative regulation of cell proliferation", the terms "response to stimulus" and "response to stress" were called as significantly enriched biological processes. Some of the genes involved in the response to stress were also implicated in inflammatory response such as FOS; IL1B; IL8; PRDX5; PROCR and TNFAI.

### Role of IL-1beta in senescence development

In apoptotic DBTRG-05 cells we did not find any significant modulation of IL1-beta or other genes involved in inflammation, while IL-1beta over-expression was observed in U87-MG cells, at the latest time points (48 h and 72 h) of the time-course.

**Figure 7:**
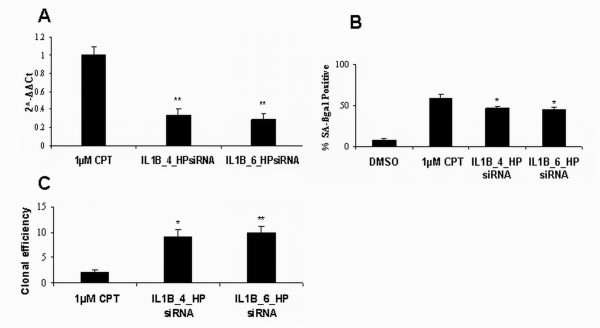
**Effects of IL-1beta gene silencing on CPT-induced senescence in U87-MG cells**. **A**. Real- time analysis of IL-1beta gene silencing. IL-1beta mRNA levels were significantly reduced (** p < 0.01, Student's t-test) with respect to mRNA level of the non silenced CPT-treated cells. IL1-B relative expression was obtained by performing the comparative method [48]. Firstly, data were normalized to GAPDH, then to a calibrator, consisting of mRNA obtained from CPT treated cells. The results were expressed as 2^^-ΔΔCt^, where ΔCt of each sample was defined as Ct_(target gene = IL1-beta) _- Ct_GAPDH, _and ΔΔCt = ΔCt_(sample = cDNA from CPT+iRNAs treated cells) _- ΔCt calibrator_(cDNA from CPT treated cells)_. ΔΔCt calibrator is always equal to 0 so that 2^^-ΔΔCt ^is always 1. **B. **SA-β gal activity, in response to 1 μM CPT, was quantified in U87-MG cells in the presence or in the absence of IL-1beta gene silencing. DMSO, solvent control. Bars, standard error. * p < 0.05, Student's t-test. **C. **250 cells from either CPT and solvent exposed cells or IL-1beta-silenced/CPT-treated U87-MG cells were sub-cultured in fresh medium in five replicates. Cells were maintained in culture for 10 days, with bi-weekly medium changes, then were fixed with methanol, stained with 10% aqueous Giemsa and scored for colony formation. Only colonies containing more than 50 cells were counted. Clonal efficiency was calculated as the mean number of colonies per plate with respect to the solvent. Bars, standard error. * p < 0.05, Student's t-test. ** p < 0.01, Student's t-test.

Taking into account that U87-MG resistance to CPT was previously suggested to be sustained by IL1-beta up-regulation [19], we decided to investigate the role that the late expression of IL-1beta could have in the development of senescence. For this purpose, U87-MG cells were transiently transfected with two different IL-1beta siRNA (IL1B_4_HPsiRNA and IL1B_6_HPsiRNA, Qiagen) and, after one day, treated with 1 μM CPT for 72 h. Both siRNAs were able to significantly knock down the expression of IL-1beta, as determined by Real-Time PCR (Figure 7A). The effect of IL-1beta silencing on the development of accelerated senescence was evaluated by measuring the SA-β-gal activity and the cloning efficiency of transfected cells (Figure 7B and 7C).

## Discussion

We adopted oligo-microarray technology to compare the transcriptional response induced by CPT-dependent DNA damage in two high-grade glioblastoma cell lines. Following TopoI inhibition, apoptosis and senescence were the different fates of DBTRG-05 and U87-MG cells, respectively. Some lines of evidence suggested that, in the presence of wild-type p53 and low CPT doses, cells mainly develop a senescence-like phenotype whereas undergo apoptosis, if p53 is mutated [5]. Taking into account that the two glioblastoma cell lines we used are both wild type for p53 [26], we suggested that the mutational status of p53 is not sufficient by itself to predict the CPT effects in our cell model. The difference in p21 protein stability between U87-MG and DBTRG-05 cells, already described by Li and coworkers [26], probably plays a more important role than the mutational status of p53 in the determination of the cell fate, senescence versus apoptosis. This results, moreover, agrees with the data reported for p53 wild-type HCT166 colon carcinoma cells, in which the ability of low doses of CPT to induce senescence and actively inhibit apoptosis was correlated to the sustained expression of p21 [4].

The overall transcriptional response, activated through a 72-hours time-course treatment with CPT, reflected, in part, the divergent biological effects triggered by TopoI inhibition. In fact, the number of genes, whose expression was affected, was much higher in the cells undergoing senescence rather than apoptosis. In addition, the disproportion between down- and up-regulated genes suggested that the development of senescence was accompanied by a general inhibition of transcription, which seemed not to affect, in a similar manner, the apoptotic DBTRG-05 cell population. Interestingly "transcription from Pol II promoter" was, indeed, one of the biological process identified by EASE analysis as significantly enriched in the list of U87-MG down-regulated genes, where we found the repression of many transcription factors mRNAs such as AHR; CHD1; CNOT2; DEK; ELL2; EPAS1; ETV1; FOXC2; FOXM1; GLI3; GTF2F2; HIPK2; HIRA; ID1; MNAT1; MYC; NCOA6; NCOR1; NR2F2; PBX3; RB1; RBBP8; SMARCA5; TCF4; TFDP1 and TRIP13.

In spite of the difference in the transcriptional profiles of the two cell lines, the general picture of the biological processes, returned by High-Troughput GoMINER and expressed in terms of GO categories, that resulted as mostly affected along the exposure time, highlighted the similarities between the two data-sets. This could arise from a general response to genotoxic stress induced by CPT, independently from the fate followed by the damaged cells (senescence or apoptosis). In fact, 60% of the genes, that were modulated in apoptotic DBTRG-05 cells, were regulated in senescent U87-MG cells, as well, and, within this list, many genes were described to be induced by DNA damage, as transcriptional targets of wild-type p53, including: p21/waf1, GADD45A, ATF3, PIG3, P53CSV, FDXR, DDIT3, KFL5 and BIRC5 [27]. In particular, a recent report has shown that BIRC5 (survivin), which inhibits apoptosis and controls cell division [28], was up-regulated in many brain cancers [29] but repressed both *in vitro *and *in vivo*, following TopoI inhibition in myeloid leukemia cells [15]. Noteworthy, regardless of the outcome due to CPT treatment, BIRC5 was down-regulated both in U87-MG and DBTRG-05 cells, confirming the relevance of this transcriptional target in the action of this drug agent.

Most of the common repressed genes were involved in DNA replication, mitosis and spindle organization. The delayed expression of mitosis related genes was already described in HCT116 non-apoptotic colon cancer cells, treated with CPT [13], and this modulation was reported as a common hallmark of the transcriptional effect induced by the activation of the DNA damage G2-checkpoint [30]. However, as highlighted by High-Throughput GoMINER and EASE functional analysis, the mitotic down-regulation induced by CPT was much more evident in senescent U87-MG cells where, in particular, the inhibition of several cyclins D1, E1, A2, B1 and B2, was accompanied by the ipo-expression of genes belonging to the mitotic spindle checkpoint pathway, such as MAD2, BUB1 and BUB3. The spindle checkpoint function monitors the completion of the spindle-kinetochore attachment and is a crucial factor to ensure the accurate sister chromatid segregation during cell division. In agreement with spindle checkpoint dysfunction, we, indeed, observed a slight increase of aneuploidy in U87-MG cells at the latest time point. Our observation is consistent with previous reports demonstrating that the down-regulation of spindle checkpoint proteins preceded the induction of senescence in human hepatoma cells exposed to a low dose of doxorubicin [6] and that normal human diploid fibroblasts underwent premature senescence in culture when BUB 1 was silenced by RNAi [31]. Taken together with other data, demonstrating that spindle checkpoint compromised HCT116-MAD2+/-cells, efficiently escaped from TopoI poison-induced apoptosis [32] and that MAD2-silenced gastric cancer SGC7901 cells increased the resistance to DNA damaging agents by up-regulating Bcl-2 [33], it is reasonable to speculate that the induction of senescence and the inhibition of apoptosis might be linked to the ipo-expression of spindle checkpoint proteins and that these proteins might also modulate the sensibility to DNA damaging agents, such as TopoI inhibitors.

In U87-MG but not in apoptotic DBTRG-05 cells, CPT treatment also led to the down-regulation of TFDP1 (DRTF1 polypeptyde 1, DP1), a transcription factor that plays a crucial role in cell cycle control, by forming a complex with the E2Fs proteins family [34]. The depletion of DP1 protein was recently reported to efficiently block E2F transcriptional activity and trigger a senescence-like cell cycle arrest [35]. Many of the genes involved in DNA replication and mitosis that were down-regulated in U87-MG cells are indeed known as E2F target genes (TK1, RRM1, RRM2, MCM3, MCM7, TOP2A, FEN1, RAD51, CDC2, CyclinA2, CyclinB1, CyclinB2, BUB1, HMG2, Stathmin, AnnexinVIII, Ki-67, RB etc) [36], suggesting a relevant role of this pathway in the development of a senescent phenotype in U87-MG cells.

Among the genes that showed a different CPT-time-dependent profile in the two cell lines, MYC was, by far, the most important one, being involved in the regulation of fundamental biological processes, such as apoptosis and cell growth. In agreement with our evidence of MYC induction in DBTRG-05 apoptotic cells, the over-expression of MYC was already described to sensitize colon cancer cells to CPT-induced apoptosis [38] and to be necessary, in rat fibroblasts, for DNA damage-initiated apoptosis in the G2 phase of the cell cycle [39]. On the other hand, the down-regulation of MYC in U87-MG cells, fits the result of a recently published paper where the suppression of MYC oncogene triggers cellular senescence, in diverse tumor types including hepatocellular carcinoma, osteosarcoma and lymphoma [40].

In a previous paper [19], we have already reported the transcriptional profile of a CPT-resistant sub-line U87CPT-R, selected through repeated cycles of high dose (15 μM) CPT treatments, and we demonstrated that these cells over-produced IL-1beta, as well as other pro-inflammatory cytokines. We also found IL1-beta induction in senescent U87-MG cells obtained after three months of continuous culturing with gradually increasing of CPT concentrations (from 1 nM to 100 nM), thus demonstrating that the transcriptional induction of IL-1beta was a common hallmark of prolonged CPT treatment. In the present time-course study, the "late" (after 48h-treatment) induction of IL-1beta by CPT, together with other proteins known to be involved in regulation of immune response and/or response to stress, was confirmed in U87-MG cells but not in the apoptotic DBTRG-05 cell line. This data suggest that the positive modulation of this cytokine might have a strict correlation with the development and maintenance of a senescent phenotype.

This hypothesis is strengthened not only by the general consideration that the inflammatory response accompanies the aging process [41] but also by the finding that the transcriptional analysis of senescent fibroblasts showed the up-regulation of IL-1beta mRNA [42].

To demonstrate a direct link between IL-1beta and the phenotype observed in CPT- treated U87-MG cells, we used a gene silencing approach. The results showed that, in spite of an efficient knocking down of the IL-1beta expression, silenced U87-MG still developed senescence, after a 72 h CPT-treatment, thus suggesting that IL-1beta had only a marginal role in senescence development and maintenance.

This observation raises the question about the real significance that IL-1beta over-production and the following burst in inflammatory response might have, mostly in relation to prolonged treatment with TopoI inhibitors. In fact the persistent production of pro-inflammatory cytokines by senescent cells, that remain metabolically active at tumor site, might actually reduce the efficacy of CPT. It is, indeed, well accepted that inflammation had a great impact on the stimulation of tumor-growth, invasiveness and angiogenesis [43] and that the over-expression of IL-1beta by the tumor microenvironment favors the process of carcinogenesis and strengthens invasiveness of already existing malignant cells [44,45], thus clearly influencing the outcome of anticancer therapy.

## Conclusion

Our data provided new insight into the transcriptional response programs induced by CPT in a cell-culture based glioblastoma model and showed that, besides a great difference in the effector program triggered by CPT (senescence vs. apoptosis), we can detect a common transcriptional signature representative of the early cellular response to CPT-induced genotoxic stress. Nevertheless, we observed that several gene expression changes account for the divergent biological effects induced by CPT, in our model. The down-regulation of MAD2, BUB1 and BUB3 mitotic spindle checkpoint proteins together with the repression of the transcription factors DP1 and MYC were suggested as key elements in the regulation of drug-mediated senescence in U87-MG cells. In addition, by analyzing the role of IL-beta 1 up-regulation in senescent cells, we opened new insights into the importance that inflammation might have in the determination of chemotherapy outcome.

In conclusion, our transcriptional analysis contributes to the search for potential biomarkers of CPT response in glioblastoma cells and add information to define the outcome of anticancer therapy in this model system.

## Methods

### Cell lines

U87-MG and DBTRG-05 glioblastoma cell lines were purchased from the American Type Culture Collection (Manassas, VA), and grown in a humidified atmosphere of 5% CO_2 _at 37°C in MEM culture medium (Invitrogen, Carlsbad, CA) supplemented with 10% Fetal Bovine Serum, 2 mM L-glutamine, 1 mM sodium pyruvate, 17.8 mM sodium bicarbonate, 0.1 mM non-essential aminoacids and in RPMI culture medium supplemented with 10% Fetal Bovine Serum and 2 mM L-glutamine (Invitrogen, Carlsbad, CA), respectively. S-(+)-camptothecin was purchased from Sigma (St. Louis, MO) and dissolved to a concentration of 3 mM in DMSO.

### Acute cytotoxicity assay

The concentration of CPT that induced 50% inhibition of cell growth (GI_50_) was determined by staining cells with crystal violet according to the protocol described previously (19). Cells were seeded at 2 × 10^5 ^cells per plate, in 60 mm Ø plates, allowed to attach for 24 h and subsequently exposed to CPT. One plate in triplicate was fixed before CPT treatment in order to measure the cell population at the time of drug addition (Tz). The GI50 was calculated according to the protocol used in the "*In vitro *Anticancer Discovery screen program" of the National Cancer Institute .

### Annexin-V apoptosis assay

To evaluate the percentage of apoptotic and necrotic cells, after a 24 h treatment with CPT, 5 × 10^4 ^cells were detached from the plates and incubated with 50 μl of labeling solution containing Annexin-V-FLUOS reagent (Roche Diagnostics GmbH, Mannheim, Germany) diluted in Hepes buffer and 1 μg/ml propidium iodide (Sigma), for 10–15 minutes at room temperature. The cells were then counted under fluorescence.

### BrdU labeling and Flow cytometric analysis

Adherent cells were pulsed with 30 μM BrdU (Sigma) for 30 min. at 37°C. After two washing steps with 1% BSA/PBS cells were fixed in 5 ml 70% ethanol for 30 min and then centrifuged at 1500 × g for 10 min. Single stranded DNA was produced by incubating fixed cells with 2N HCl/Triton X100 for 30 min at room temperature. After two washing steps, 10^6 ^cells were re-suspended in 1 ml of 0.5%Tween 20/1% BSA/PBS and incubated with 20 μl of Anti-BrdU FITC (Becton Dickinson, San Josè, CA) for 30 min at room temperature. Washed cells were then re-suspended in 1 ml of PBS containing 50 μg/ml propidium iodide. The analysis of DNA content and cell cycle were performed using a FACSCalibur equipped with 488 nm laser (Becton Dickinson, San Jose, CA, USA) and Cells Quest software (10.000 cells analyzed for each sample).

### SA-β-Gal staining

To detect senescence – associated – β-galactosidase (SA-β-gal) activity, cells were washed twice in PBS and fixed in a buffer containing 2% formaldehyde and 0.2% glutaraldehyde in PBS for 5 min. at room temperature (Sigma). SA-β-gal staining was then performed using a citric acid/phosphate buffer (pH 6.00) containing 1 mg/ml of X-Gal, 5 mM potassium ferricyanide, 5 mM potassium ferrocyanide, 2 mM MgCl_2 _and 150 mM NaCl (Sigma). A blue color was visible in senescent cells within 2 h but reached the maximum between 12 to 16 h. At that time, counts were made on three random fields of about 100 cells.

### RNA preparation and hybridization

Total RNA was isolated from exponentially growing cells using TRIzol Reagent (Life Technologies, Carlsbad, CA) and purified on Rneasy^R ^affinity column (Qiagen, Valencia, CA). The quality of RNA was assessed with the Agilent bioanalyzer 2100 using the RNA nano kit (Agilent Technologies, Palo Alto, CA). cDNA was synthesised from 20 μg of total RNA and labelled with Cy3-dCTP or Cy5-dCTP (Perkin-Elmer, Boston, MA) following in details the manufacturer protocol (Agilent fluorescent direct label kit, G2555-98003, version 2.1. available online at ), optimized for use with Agilent oligo-microarray Kit. Labelled cDNA from the two reactions was combined, purified with QIAquick spin column (Qiagen), and then applied to the oligonucleotide slide (Human1A oligo microarray, G4110A) according to the Agilent 60-mer oligo microarray processing protocol (G4140-90010 version 7.1. available online at ). Slides were scanned in both Cy-3 and Cy-5 channels with Agilent dual laser microarray scanner (G2565AA). Scanned images were analyzed by the Agilent Feature Extraction software 7.5 to derive the raw intensity data used in the next steps of analysis. The raw data discussed in this publication have been deposited in Arrayexpress, the EBI microarray data public repository (Arrayexpress ). The U87-MG has been assigned the accession number: E-MEXP-741. The DBTRG- has been assigned the accession number: E-MEXP-751.

### Experimental design and microarray data analysis

For each cell line we adopted an experimental design consisting of six after-treatment time points (2 h, 6 h, 16 h, 24 h, 48 h, 72 h) compared to an untreated zero-time common reference. Total RNA samples, corresponding to each time point, were derived from a pool of RNAs extracted from three independent experiments. Each time point was replicated by performing a dye-swap. The data-sets of the two time-series experiment underwent preliminary data filtering procedures. Data, corresponding to features flagged as controls, were filtered out before proceeding to the following analysis. Filtered raw (median green and red signal) intensity (17874 features for the "U87_CPT" and 17905 features for the "DBTRG_CPT") were then log_2_-tranformed and normalized intra-array for intensity- and position-dependent bias using the Joint-LOWESS algorithm.

To assess differentially expressed genes, the transformed data were analyzed by using MAANOVA (Micro Array ANalysis Of VAriance) data analysis package of R programming environments [23]. The fixed-effect linear ANOVA model, *y*_ijkg _= *μ *+ *A*_*i *_+ *D*_*j *_+ *T*_*k *_+ *G*_*g *_+ *AG*_*ig *_+ *DG*_*jg *_+ *TG*_*kg *_+ *ε*_*ijkg*_, was chosen to fit transformed intensity data *y*, where *μ *is the overall mean expression level and *ε*_*ijkg *_is the residual measurement effect. This model allows you to take into account the different sources of variance of an experiment due to array (*A*), dye (*D*), gene (*G*), time points (*T*) and to their combined effects (*AG*, *DG *and *TG*). The term *TG *is that of primary interest in our analysis; it captures variations in the expression levels of a gene across the time points.

We then tested a null hypothesis of no differential expression (so that all *TG *values are equal to zero) using F statistics computed on the James-Stein shrinkage estimates of the error variance [46]. To avoid any assumption on error distribution, the package offers the possibility of computing p-values for hypothesis tests via permutation methods (in our analyzes 1000 permutations with sample shuffling were carried out). Finally the false-discovery rate controlling method [47] was used to correct significance estimate for multiple testing hypothesis. In the analysis of the "U87_CPT" we selected, as differentially expressed, the features with p < 0.01 in the F test, after false-discovery rate adjustment, while in the analysis of the "DBTRG_CPT", the chosen threshold p-value was 0.05. Therefore, 3168 and 155 genes were deemed as significantly modulated in the "U87_CPT" and the "DBTRG_CPT" analysis, respectively.

### High-throughput GoMiner and EASE analysis of microarray data

High-throughput GoMiner, calculates for each category the enrichment factor Re = (nf/n)/(Nf/N), where nf is the number of flagged genes within the category (i.e., genes whose expression levels are considered to be changed beyond a given threshold), n is the total number of genes within that same category, Nf is the number of flagged genes on the entire microarray, and N is the total number of genes on the microarray. For each category a Fisher's exact p-values (for the one-tailed test) was calculated to measure its statistical significance. Furthermore to address the question of multiple comparisons the program also estimates the False discovery rate (FDR) by using a re-sampling algorithm.

EASE first calculates the List Hits (number of genes in the gene list that belong to the Gene Category), the List Total (number of genes in the gene list), the Population Hits (number of genes in the total group of genes assayed that belong to the specific Gene Category) and the Population Total (number of genes in the total group of genes assayed that belong to any Gene category within the system). Then the probability of seeing the number of "List Hits" in the "List Total" given the frequency of "Population Hits" in the "Population Total" is calculated as the Fisher exact probability. EASE also calculate another metric known as the "EASE score" which is the upper bound of the distribution of Jackknife Fisher exact probabilities. The EASE score is a conservative adjustment of the Fisher exact that strongly penalizes the significance of categories supported by few genes. Moreover in order to address the multiple comparisons problem we used Bonferroni method to correct the EASE score.

### Semi-quantitative RT-PCR

Some gene expression changes detected in the microarray were validated by semi-quantitative Rt-PCR. 1 μg of total RNA was retro-transcribed to cDNA using 200 Units of SuperScript II RnaseH- Reverse Transcriptase (Life Technologies), with 25 μg/ml oligo(dT) primer and 500 μM dNTP mix.

PCR was performed with a reduced number of cycles, using less than 10% of the RT reaction, with 5 Units of Taq DNA polymerase (Life Technologies), 1.5 μM MgCl2, 200 μM dNTP mix and 20 μM forward/reverse primers for: IL-1ß (5'gggcctcaaggaaaagaatc3'/5'ttctgcttgagaggtgctga3'), EREG (5'cctggtgcacagtgcttaga3'/5'actccccagggttagcttgt3'), GEM (5'gggagagagtgggagtttcc3'/5'aaagatgttggccagagtgg3'), MYC (5'ctcctggcaaaaggtcagag3'/5'ggccttttcattgttttcca3'), TXNIP (5'gccacacttaccttgccaat3'/5'ggaggagcttctggggtatc3'), AREG (5'tggattggacctcaatgaca3'/5'ccatttttgcctcccttttt3'), GADD45(5'ggaggaagtgctcagcaaag3'/5'tcccggcaaaaacaaataag3'), CDKN1A (5'gacaccactggagggtgact3'/5'tggattagggcttcctcttgg3') (Sigma). Reactions were run on GeneAmp PCR System 9600 (Applied Biosystems Foster City, USA).

The amplified fragments were analyzed and quantified using the DNA1000 kit of the Agilent 2100 Bioanalyzer.

### siRNA and quantitative real-time PCR

We used two different HP GenomeWide siRNA duplexes against IL-1beta (IL1B_4_HPsiRNA and IL1B_6_HPsiRNA, Qiagen). U87-MG cells were transfected with HiPerFect Trasfection Reagent (Qiagen) according to the manufacturer's protocol, using a final concentration of 10 nM siRNA. The transfection efficiency was tested by a non-silencing control siRNA, labeled with Alexa Fluor 488 (Qiagen). Gene silencing was checked by real-time RT-PCR using the SYBR Green qPCR Supermix (Invitrogen) and two different forward/reverse primers for IL-1beta (a: 5'aaacctcttcgaggcacaag3'/5'ctgtttagggccatcagctt3'; b: 5'gggcctcaaggaaaagaatc3'/5'ttctgcttgagaggtgctga3'), depending on the siRNA target sequence.

Total RNA was extracted using TriZol reagent and purified on RNeasy^R ^mini affinity column kit (Qiagen), and cDNA was synthesized with the SuperScript™ III First-Strand Synthesis SuperMix (Invitrogen). The level of test cDNA relative to that of GAPDH was calculated by the $2^{-\Delta \Delta {\text{C}}_{\text{T}}}$ method. [46]

## Competing interests

The authors declare that they have no competing interests.

## Authors' contributions

EM planned and performed gene expression profiling laboratory work and data analysis, carried out siRNA experiments and prepared the draft version of the manuscript. CS planned and performed gene expression profiling laboratory work and data analysis, carried out siRNA experiments, and prepared the draft version of the manuscript. DQ performed statistical analysis. GD was involved in the first steps of statistical data analysis, SP performed RT-PCR experimental validation. MC and GF participated in FACS analysis. MM carried out the cell culture treatments and cell viability assessments. WH and MV participated in the study design and in the scientific discussion. RS contributed to the conception of the study. AC performed the supervision and coordination of the study. PS participated in the supervision of the study and manuscript preparation.

All authors have read and approved the final version of the manuscript.

## Availability & requirements









## Supplementary Material

Additional file 1 **U87_datasets of differentially expressed genes**. File contain the complete list of differentially expressed genes identified by MAANOVA. Click here for file

Additional file 2 **DBTRG_datasets of differentially expressed genes**. File contain the complete list of differentially expressed genes identified by MAANOVA. Click here for file

Additional file 3 **GoMiner Hightroughput Functional analysis of up-regulated GO categories in either U87-MG and DBTRG-05**. Clustered Image Maps (CIM) with hierarchically clustered (euclidean distance, avarage linkage clustering) up-regulated GO categories versus time. The scale corresponds to the following numerical transformation of the FDR (false discovery rate) value: T-0.9*FDR, where T is the chosen value of significance (in our analysis 0.05). Click here for file

Additional file 4 **GoMiner Hightroughput Functional analysis of down-regulated Go categories in U87-MG**. Clustered Image Maps (CIM) with hierarchically clustered (euclidean distance, avarage linkage clustering) down-regulated GO categories versus time. The scale corresponds to the following numerical transformation of the FDR (false discovery rate) value: T-0.9*FDR, where T is the chosen value of significance (in our analysis 0.05). Click here for file

Additional file 5 **GoMiner Hightroughput Functional analysis of down-regulated Go categories in DBTRG-05**. Clustered Image Maps (CIM) with hierarchically clustered (euclidean distance, avarage linkage clustering) down-regulated GO categories versus time. The scale corresponds to the following numerical transformation of the FDR (false discovery rate) value: T-0.9*FDR, where T is the chosen value of significance (in our analysis 0.05). Click here for file
