# Supplementary figures and images for: Gene expression time-series analysis of Camptothecin effects in U87-MG and DBTRG-05 glioblastoma cell lines
Source: Mol Cancer. 2008 Aug 11;7:66. doi: 10.1186/1476-4598-7-66 (PMC2556695; doi:10.1186/1476-4598-7-66)

## Slide 1
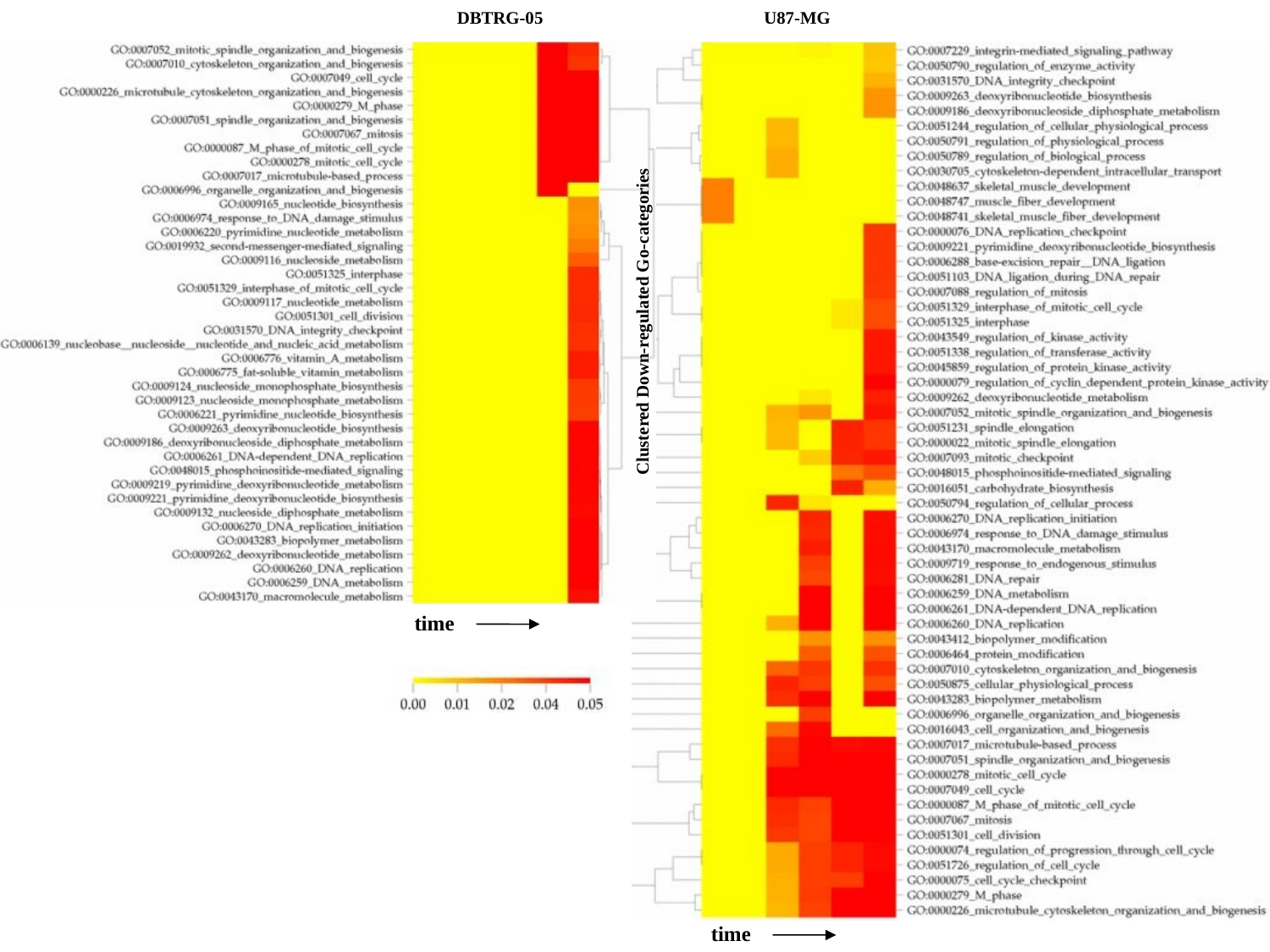

DBTRG-05
U87-MG
Clustered Down-regulated Go-categories
time
time

Supplement: Additional file 3 — GoMiner Hightroughput Functional analysis of up-regulated GO categories in either U87-MG and DBTRG-05. Clustered Image Maps (CIM) with hierarchically clustered (euclidean distance, avarage linkage clustering) up-regulated GO categories versus time. The scale corresponds to the following numerical transformation of the FDR (false discovery rate) value: T-0.9*FDR, where T is the chosen value of significance (in our analysis 0.05). [file 1476-4598-7-66-S3.ppt]

## Slide 1
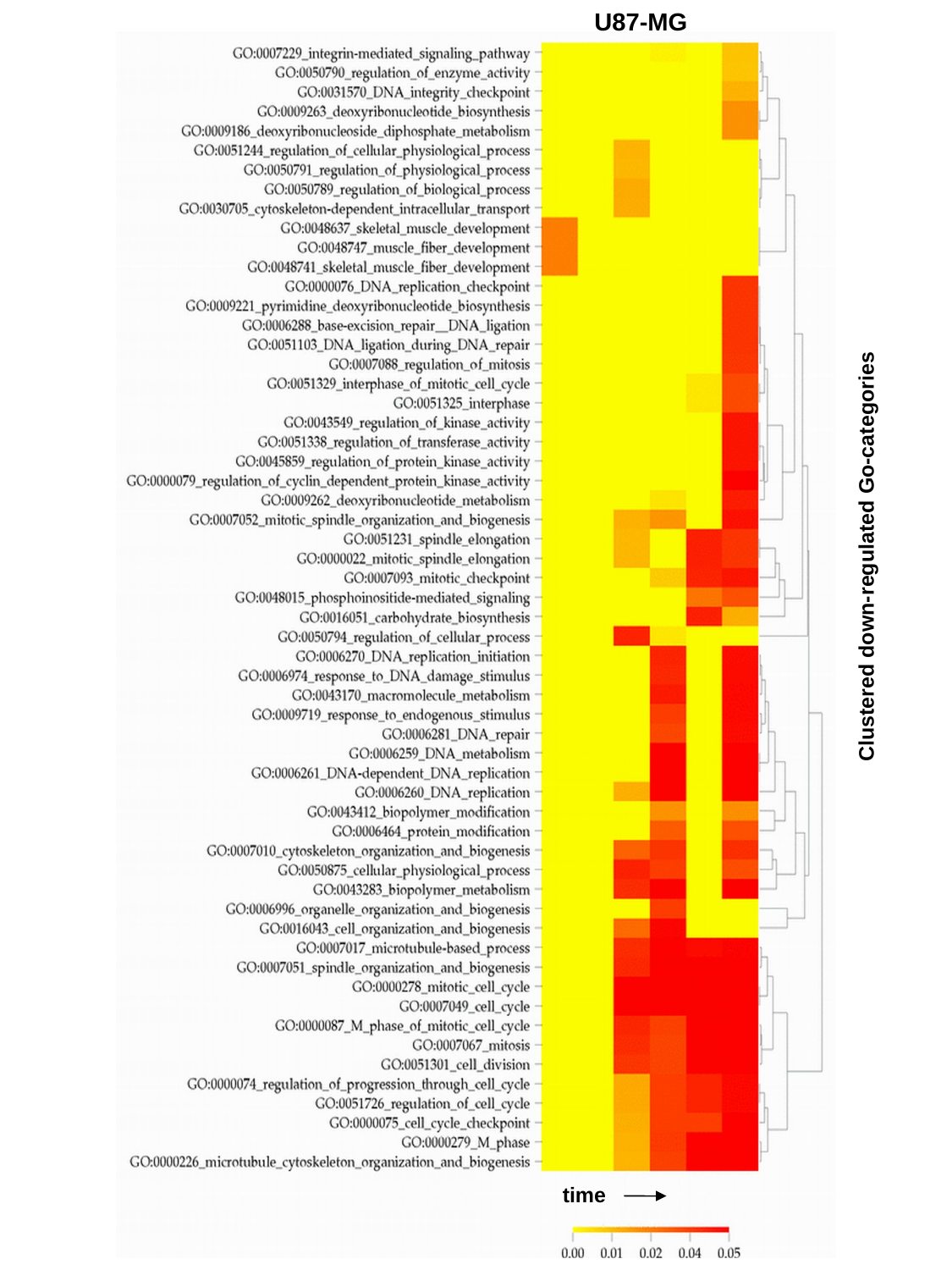

U87-MG
Clustered down-regulated Go-categories
time

Supplement: Additional file 4 — GoMiner Hightroughput Functional analysis of down-regulated Go categories in U87-MG. Clustered Image Maps (CIM) with hierarchically clustered (euclidean distance, avarage linkage clustering) down-regulated GO categories versus time. The scale corresponds to the following numerical transformation of the FDR (false discovery rate) value: T-0.9*FDR, where T is the chosen value of significance (in our analysis 0.05). [file 1476-4598-7-66-S4.ppt]

## Slide 1
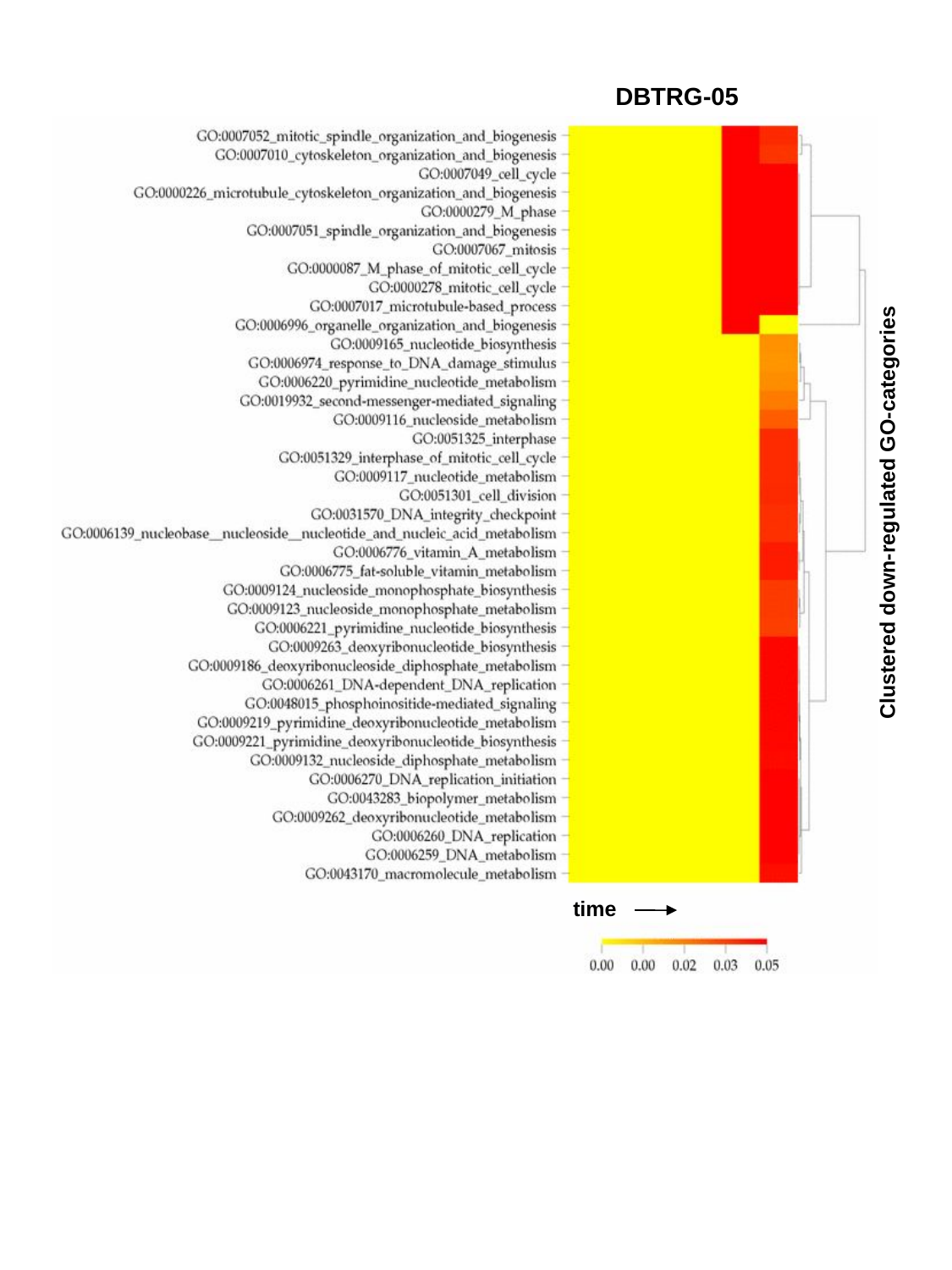

DBTRG-05
Clustered down-regulated GO-categories
time

Supplement: Additional file 5 — GoMiner Hightroughput Functional analysis of down-regulated Go categories in DBTRG-05. Clustered Image Maps (CIM) with hierarchically clustered (euclidean distance, avarage linkage clustering) down-regulated GO categories versus time. The scale corresponds to the following numerical transformation of the FDR (false discovery rate) value: T-0.9*FDR, where T is the chosen value of significance (in our analysis 0.05). [file 1476-4598-7-66-S5.ppt]
